# The Successful Resolution of a Large Splenic Abscess With Six Years of Follow-Up and Without Recurrence

**DOI:** 10.7759/cureus.53042

**Published:** 2024-01-27

**Authors:** Afnan D Almasaud, Ihab F Sulaiman

**Affiliations:** 1 College of Medicine, King Saud Bin Abdulaziz University for Health Sciences, Riyadh, SAU; 2 Cardiology, Division of Adult Cardiology and Advanced Cardiac Imaging, King Abdulaziz Cardiac Center, King Abdulaziz Medical City, Ministry of National Guard Health Affairs, Riyadh, SAU

**Keywords:** multilocular abscess, radiology interventional, virtual follow-up, ultrasound-guided drianage, interventional radiology guided drainage, spleen abscess

## Abstract

Splenic abscess is an uncommon medical condition that presents many diagnostic difficulties. Although rare, this clinical disease can be potentially life-threatening, with a fatality rate exceeding 70% in immunocompromised patients. Clinical manifestations of splenic abscess include fever, tenderness in the upper left region of the abdomen, and leukocytosis with left shift. Performing abdominal ultrasonography (USG) and CT in patients presenting with clinical manifestations enables a prompt and accurate diagnosis. The treatment for splenic abscess includes medical therapy, imaging-guided drainage, and splenectomy. We discuss the case of a 60-year-old female patient who presented to our emergency department with a large splenic abscess; she was managed with an ultrasound-guided drainage catheter, which led to the successful resolution of the condition. She did not experience any recurrence during six years of follow-up. This case report aims to highlight the role of interventional radiology in managing splenic abscesses.

## Introduction

Splenic abscess is a rare medical condition with an incidence of 0.14-0.70%. In the past, up to 60% of patients with splenic abscesses succumbed to the condition due to difficulties in symptom recognition and late diagnosis [1]. The risk factors for splenic abscess include metastatic infection, diabetes mellitus, trauma, malignancy, and congenital or acquired immunodeficiency [2,3]. It commonly arises as a consequence of infectious endocarditis, affecting approximately 5% of patients [4]. The most commonly encountered infections in isolation involve aerobic bacteria, such as Streptococcus, Staphylococcus (since endocarditis is the primary cause of splenic abscess), and Enterococcus [5]. Other reported organisms include Mycobacterium, and fungi such as Candida, Aspergillus, and Blastomyces [5]. In melioidosis-endemic areas, Burkholderia pseudomallei is the most common organism associated with splenic abscesses [4].

The clinical manifestations of splenic abscess usually include upper left abdominal pain, along with fever, fatigue, and vomiting. Laboratory workup will mostly reveal leukocytosis with left shift [4]. The treatment of splenic abscess involves a combination of medical therapy with antibiotics and imaging-guided drainage or surgical intervention like splenectomy [6]. The mortality rates in spleen abscesses are high and depend on the patient's immunocompetency and the type of acquired infection. The fatality rate of spleen abscesses in immunocompromised patients with multilocular abscesses can be as high as 80% while that in immunocompetent patients with unilocular abscesses reaches up to 15% [4].

## Case presentation

The patient was a 60-year-old female with diabetes, chronic kidney disease, and hypertension who presented to our emergency department with abdominal distension, nausea, and vomiting for five days. She also reported complete constipation for two weeks. The patient did not experience any symptoms of fever, chills, or shivering. Moreover, there was no jaundice, melena, urinary symptoms, weight loss, and no history of trauma. Upon initial assessment, the patient was tachycardic and tachypneic. The abdominal examination revealed a distended and tympanic abdomen, with marked tenderness localized to the left upper quadrant. No abnormalities were found in a further systemic examination.

**Figure 1:**
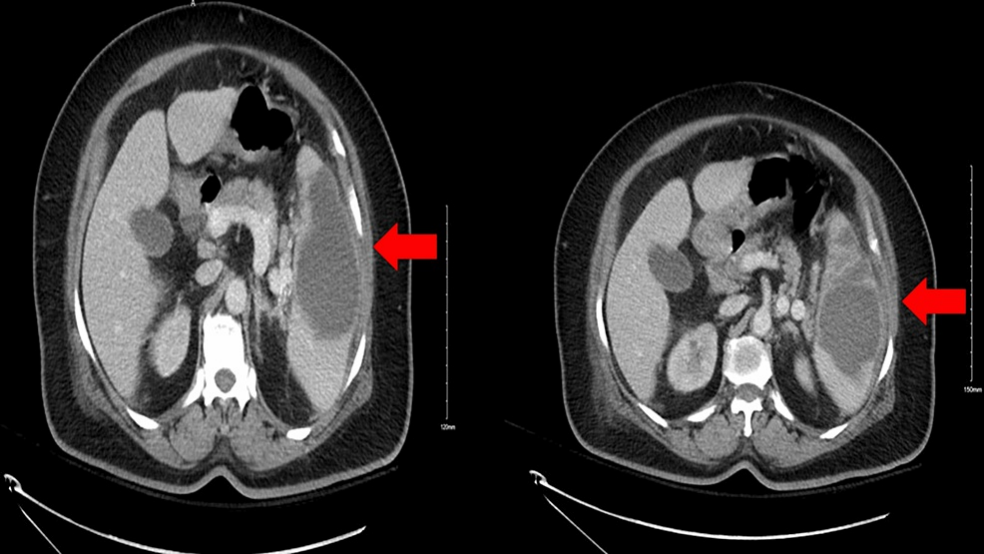
Contrast-enhanced CT of the abdomen upon admission demonstrating a multiloculated collection (red arrows) CT: computed tomography

The laboratory studies revealed a markedly increased white blood cell count of 20,700 per μl (normal range: 4,500-11,000 WBCs per μl) with left shift, a significant increase in lactic acid levels, and hyperglycemia. The coagulation profile was normal. Blood and urine cultures were performed and showed no growth. HIV and tuberculosis screening were negative. A CT scan was performed and showed a multiloculated collection measuring 9.3 x 7.9 cm with subcapsular fluid (Figure 1). To exclude the possibility of infectious endocarditis, transesophageal echocardiography was performed and revealed normal ejection fraction and normal mitral valve function and structure.

The diagnosis of splenic abscess was established based on CT scan results, and the patient was promptly started on ampicillin sulbactam. Under local anesthesia, ultrasound-guided drainage was placed in the splenic collection, and about 20 ml of dark bloody pus was aspirated and sent for laboratory investigation. The patient tolerated the procedure without any complications. The cultures obtained from the drainage catheter exhibited heavy growth of Staphylococcus aureus, with no anaerobic organisms. According to the culture and sensitivity data, the antibiotic treatment was changed to cefazolin. 

**Figure 2:**
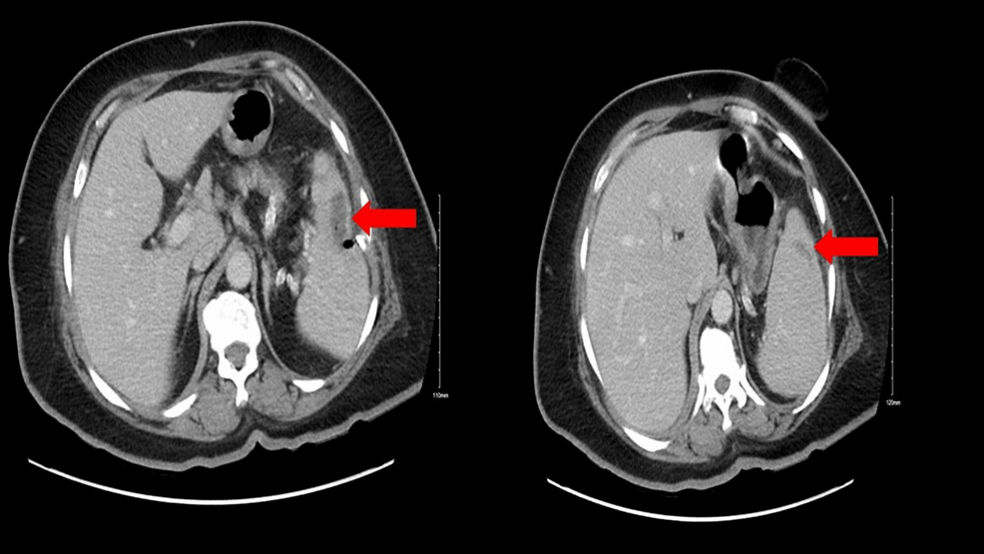
Follow-up CT scan performed six months after the discharge showing almost-complete resolution of the splenic abscess (red arrows) CT: computed tomography

A subsequent CT scan eight days after the drainage procedure revealed significant improvement in the patient's condition. The size of the splenic collection had diminished, and the patient was discharged home with outpatient follow-up. At the one-month follow-up, the patient remained asymptomatic. A CT scan performed within six months of follow-up revealed almost full recovery (Figure 2). Also, a virtual follow-up conducted after six years revealed no indications of recurrence or any newly emerging issues.

## Discussion

Splenic abscesses are most commonly caused by metastatic hematogenous infections related to endocarditis, contiguous infection sites, or trauma [1]. Lifestyle changes worldwide have increased the prevalence of diabetes mellitus, malignancies, and immunosuppression [1]. These underlying factors increase the probability of developing a splenic abscess. A retrospective examination of 67 cases of splenic abscesses by Chang et al. found that 54 of these cases were linked to an underlying predisposing disease [6]. Of these, diabetes mellitus was identified as the main contributing factor [1]. In addition, Sreekar et al. have provided additional evidence through a retrospective review of 75 instances of splenic abscess [7]. Out of 75 patients presenting with splenic abscess, diabetes mellitus was present in 20 [7]. If a splenic abscess is found, it is very important to search for underlying diseases that might have caused the abscess formation. These include other infections (such as endocarditis), diabetes, malignancies, and immunodeficiency diseases [5]. Our case had uncontrolled type II diabetes, hypertension, and chronic kidney diseases. Moreover, infectious endocarditis was excluded based on transesophageal echocardiography results.

Splenic abscesses usually present with nonspecific signs and symptoms, such as fever, fatigue, left hypochondrium pain, and marked leukocytosis with a left shift [5]. They are often misdiagnosed because the signs and symptoms are vague or similar to other diseases of the spleen like hematoma or malignant lesions. In most studies, the commonly reported microorganisms are aerobic bacteria such as Streptococcus, Staphylococcus, and Enterococcus [1]. Also, cases involving facultative anaerobic bacteria like Escherichia coli have been described. A team from Spain found that 36.4% of the 22 cases they studied were caused by Mycobacterium tuberculosis [8]. The study by two scientists from Japan and one researcher in Taiwan found that bacteria called gram-negative rods accounted for 46.7-60% of the cases they analyzed [6]. Our patient presented with left hypochondrium pain, and there was abdominal distension with left hypochondrium tenderness upon abdominal examination. The laboratory evaluation revealed leukocytosis and the drainage culture revealed heavy growth of Staphylococcus aureus.

Detecting a splenic abscess can be challenging. The laboratory evaluation usually reveals acute phase reactants. Radiological studies like X-rays and ultrasound might be helpful for the initial assessment of a splenic abscess [4]. CT scan is considered the best diagnostic tool to detect splenic abscesses [4]. Percutaneous drainage guided by ultrasound or CT scan can be valuable both diagnostically and therapeutically [4]. Historically, antibiotics and splenectomy have been considered the most effective and widely accepted treatment options for patients diagnosed with a splenic abscess [9]. However, our patient was deemed unsuitable for surgical intervention due to multiple comorbidities. Exclusively relying on antibiotics in the management of splenic abscesses is not advisable and continues to be a topic of debate. Patients solely treated with antibiotics have been associated with mortality rates exceeding 50% [4].

Percutaneous drainage is commonly employed for intra-abdominal abscesses. It is a safe and less invasive procedure and free from complications associated with open surgery [3]. However, there have been reports of possible complications associated with percutaneous drainage, such as fistula, pneumothorax, or bowel perforation [10]. The outlook for a splenic abscess has significantly improved nowadays compared to the past. Moreover, laparoscopic splenectomy has emerged as a highly promising alternative to the conventional open procedure, offering advantages such as accelerated healing and reduced length of hospitalization. Surgical splenectomy is considered the last resort for therapy because most patients may be effectively handled by percutaneous guided drainage and antibiotics.

Recent studies suggest that CT-guided drainage is a safe and efficient method for treating a splenic abscess [4]. If surgery is required, it is advisable to opt for a laparoscopic method rather than an open one [4]. Research has shown that the average duration of hospitalization for patients who received surgical procedures is 15.83 days [7].

## Conclusions

An abscess of the spleen is an uncommon condition that poses a significant risk to immunocompromised people and those with comorbidities and has a high morbidity and mortality rate. Splenic abscesses can manifest with nonspecific signs and symptoms or those associated with other intra-abdominal pathologies. A high index of suspension is required for a timely and accurate diagnosis. Interventional radiology is the mainstay of the treatment of spleen abscesses, given its comparable effectiveness to surgical intervention, especially in patients with multiple comorbidities who are unsuitable for surgical intervention.
